# Validation of non-invasive hemodynamic monitoring with Nexfin in critically ill patients

**DOI:** 10.1186/cc9495

**Published:** 2011-03-11

**Authors:** K Van de Vijver, A Verstraeten, C Gillebert, U Maniewski, M Gabrovska, D Viskens, N Van Regenmortel, I De laet, K Schoonheydt, H Dits, M Malbrain

**Affiliations:** 1 ZNA Stuivenberg, Antwerp, Belgium

## Introduction

Thermodilution (TD) is a gold standard for cardiac output (CO) measurement in critically ill patients [1]. Although transpulmonary thermodiluation is less invasive than the Swan-Ganz catheter, it still requires an arterial and deep venous line. This study will compare intermittent bolus transpulmonary TDCO with continuous CO (CCO) obtained by pulse contour analysis (PiCCO2; Pulsion Medical Systems) and non-invasive CO (NexCO) measurement via finger cuff using Finapres technology (Nexfin BMEYE).

## Methods

A prospective study in 45 patients (43 mechanically ventilated, 32 male). Age 57.6 ± 19.4, BMI 25.3 ± 4.4, SAPS II 51.5 ± 16.9, APACHE II 25.3 ± 10.3 and SOFA score 9.4 ± 3.3. In an 8-hour period, simultaneous CCO and NexCO measurements were obtained every 2 hours while simultaneous TDCO and NexCO were obtained every 4 hours. The CCO and NexCO values were recorded within 5 minutes before TDCO was determined. Statistical analysis was performed using Pearson correlation and Bland-Altman analysis.

## Results

In total, 585 CO values were obtained: 225 paired CCO-NexCO; 135 paired CCO-TDCO and 135 NexCO-TDCO. Thirty-five patients received norepinephrine at a dose of 0.2 ± 0.2 μg/kg/minute (range 0.02 to 1). TDCO values ranged from 2.4 to 14.9 l/minute (mean 6.6 ± 2.2), CCO ranged from 1.8 to 15.6 l/minute (6.4 ± 2.3) and NexCO from 0.8 to 14.9 l/minute (6.1 ± 2.3). The Pearson correlation coefficient comparing NexCO with TDCO and CCO was similar with an *R*^2 ^of 0.68 and 0.71 respectively. Bland-Altman analysis comparing NexCO with TDCO revealed a mean bias ± 2SD (limits of agreement (LA)) of 0.4 ± 2.32 l/minute (with 36.1% error) while analysis of NexCO versus CCO showed a bias (± LA) of 0.2 ± 2.32 l/minute (37% error). TDCO was highly correlated with CCO (*R*^2 ^= 0.95) with bias 0.2 ± 0.86 (% error 13.3). The MAP values obtained ranged from 43 to 140 mmHg (83 ± 17) for PiCCO_2 _and from 44 to 131 (85 ± 17) for Nexfin. The MAP obtained with Nexfin correlated well with invasive MAP via PiCCO2 (*R*^2 ^= 0.89) with a bias (± LA) of 2.3 ± 12.4 (% error 14.7).

## Conclusions

These preliminary results indicate that in unstable critically ill patients CO and MAP can be reliably monitored non-invasively with Nexfin technology. Although TPTD remains a gold stand for the measurement of CO in ICU patients, Nexfin non-invasive monitoring may provide useful information in the emergency or operating room when an arterial or CVL is not available.
